# Does genotypic diversity of *Hydrocotyle vulgaris* affect CO_2_ and CH_4_ fluxes?

**DOI:** 10.3389/fpls.2023.1272313

**Published:** 2023-10-09

**Authors:** Jia-Tao Zhu, Wei Xue, Jun-Qin Gao, Qian-Wei Li, Wen-Han Yu, Fei-Hai Yu

**Affiliations:** ^1^ School of Ecology and Nature Conservation, Beijing Forestry University, Beijing, China; ^2^ Institute of Wetland Ecology & Clone Ecology/Zhejiang Provincial Key Laboratory of Evolutionary Ecology and Conservation, Taizhou University, Taizhou, Zhejiang, China; ^3^ The Key Laboratory of Ecological Protection in the Yellow River Basin of National Forestry and Grassland Administration, Beijing, China

**Keywords:** clonal plant, diversity effect, genetic diversity, greenhouse gas, *Hydrocotyle vulgaris*

## Abstract

Biodiversity plays important roles in ecosystem functions and genetic diversity is a key component of biodiversity. While effects of genetic diversity on ecosystem functions have been extensively documented, no study has tested how genetic diversity of plants influences greenhouse gas fluxes from plant-soil systems. We assembled experimental populations consisting of 1, 4 or 8 genotypes of the clonal plant *Hydrocotyle vulgaris* in microcosms, and measured fluxes of CO_2_ and CH_4_ from the microcosms. The fluxes of CO_2_ and CO_2_ equivalent from the microcosms with the 1-genotype populations of *H. vulgaris* were significantly lower than those with the 4- and 8-genotype populations, and such an effect increased significantly with increasing the growth period. The cumulative CO_2_ flux was significantly negatively related to the growth of the *H. vulgaris* populations. However, genotypic diversity did not significantly affect the flux of CH_4_. We conclude that genotypic diversity of plant populations can influence CO_2_ flux from plant-soil systems. The findings highlight the importance of genetic diversity in regulating greenhouse gas fluxes.

## 1 Introduction

Greenhouse gas emissions have increased since the pre-industrial era, which is primarily driven by economic development and population increment (Stocker, 2014). Specially, CO_2_ is the largest contributor, accounting for 74.4% of the total emissions, and CH_4_ is the second largest contributor, accounting for 17.3% (Ritchie et al., 2020). Terrestrial ecosystems are the important carbon sinks of greenhouse gases that are profoundly influenced by plants, soil and other environment factors (Yuan et al., 2015). Specifically, plant species identity and diversity have a direct impact on photosynthetic carbon sequestration and an indirect influence on CO_2_ and CH_4_ emissions by altering biochemical processes (Fang, 2010; Koelbener et al., 2010).

Plant species diversity can impact ecosystem function, such as primary productivity and nutrient cycling (Cardinale et al., 2012; van der Plas, 2019). As plant species richness in a community increases, the probability of highly productive species arises (i.e., sampling effect), or resources in the community can be used more completely due to niche partition and complementarity among species (i.e., complementarity effect), thereby resulting in an increase e.g., community productivity, invasion resistance and carbon sequestration (Loreau and Hector, 2001; Adomako et al., 2019; Xue et al., 2021). Studies have shown that increasing plant species diversity is potentially able to influence greenhouse gas emissions from ecosystems (Han et al., 2019; Luo et al., 2020; Fan et al., 2021).

Besides species diversity, genetic diversity is another key component of biodiversity (Jiang et al., 2021; Begum et al., 2022; Huang et al., 2022). Similar to the impact of plant species diversity, increasing plant genetic diversity can also affect population performance and ecosystem functions (Hughes et al., 2008; Begum et al., 2022). For instance, genotypic diversity has been shown to affect plant biomass, root morphology and nutrient uptake (Abbott and Stachowicz, 2016; Semchenko et al., 2021; Huang et al., 2022; Begum et al., 2023). So far, it remains unclear whether genotypic diversity of a plant can affect greenhouse gas fluxes from plant-soil systems.

CO_2_ flux is determined by photosynthetic carbon input and plant-soil respiration emission, while CH_4_ flux is more affected by methanogens and substrate quality (Mo et al., 2015; Gao et al., 2018). The capacity of photosynthetic carbon sequestration and plant-soil respiration can vary greatly among different genotypes of the same plant (Cook-Patton et al., 2011). Additionally, different genotypes of the same plant may be associated with different soil microbial communities that may greatly impact the processes of CH_4_ production and soil respiration (Johnson et al., 2010; Latta et al., 2011; Burrill et al., 2023). Hence, we postulated that genotypic identity and genotype diversity of the same plant species may affect greenhouse gas fluxes (e.g., CO_2_ and CH_4_ fluxes).

We assembled experimental populations consisting of 1, 4 or 8 genotypes of the clonal plant *Hydrocotyle vulgaris* in microcosms, and measured fluxes of CO_2_ and CH_4_ from the microcosms. Specifically, we addressed the following two questions: (1) Does genotypic identity of *H. vulgaris* influence CO_2_ and CH_4_ fluxes from the microcosms? (2) Does genotypic diversity of *H. vulgaris* affect CO_2_ and CH_4_ fluxes from the microcosms?

## 2 Materials and methods

### 2.1 Species information and preparation

*Hydrocotyle vulgaris* L. (Araliaceae) is a perennial clonal plant with creeping stems rooted in each node in humid conditions (Dong et al., 2015). Commonly, each stem node has a leaf and a new creeping stem can be formed at the leaf axil (Si et al., 2020). It is native to Europe and the United States, often inhabiting moist habitats such as rivers, ponds, swamps, valleys, and dune grasslands (Xue et al., 2022). This species can reproduce rapidly by clonal growth and show high morphological plasticity (Wang et al., 2022). Different genotypes of *H. vulgaris* differ in competitive ability (Zhang L. et al., 2022) and clonal integration (Si et al., 2020).

In 2016, 128 ramets of *H. vulgaris* were collected from 10 different sites in China (Wang et al., 2020; Huang et al., 2022). Total genomic DNA of the ramets were extracted and a total of 20 genotypes were identified by ALFP based on genomic DNA (see Wang et al., 2020 for detail). To meet the experimental requirements and reduce the influence of genotype identity on genotypic diversity (Hughes et al., 2008; Hughes and Stachowicz, 2009), we randomly selected ten genotypes of *H. vulgaris* to construct populations with different levels of genotypic diversity. Among the ten genotypes, three were from Wenzhou, two were from Chongqing, and one was each of Taizhou, Jiangxi, Hangzhou, Wuhan and Lishui (Wang et al., 2020). Ramets of these ten genotypes were vegetatively propagated in greenhouses of Taizhou University in Taizhou, Zhejiang Province, China. On August 18, 2021, 1920 ramets (a node with a leaf and some adventitious roots) of similar size were selected. The initial height of the ramets was 4.04 ± 0.12 cm (mean ± SE, n =10), and the initial dry weight of the ramets 0.038 ± 0.003 g (mean ± SE, n =10).

### 2.2 Experimental design

Since 1, 4, and 8 genotypes are commonly used to represent the low, medium, and high genotype diversity (e.g., Hughes et al., 2008; Latta et al., 2011; Xue et al., 2021), we used these three levels of genotypic richness to construct populations. Each population was construed in a pot (16 cm in diameter and 20 cm in height) filled with a 1:1 mixture of river sand and soil collected from a wasteland in Taizhou, Zhejiang Province, China. Each pot was planted with 16 ramets of *H. vulgaris*. During the experiment, we used strings and labels to distinguish genotypes without disturbing plant growth, and we did not separate the individuals of different genotypes in the mixture plot.

For the 1-genotype populations, each pot was grown with 16 ramets of the same genotype, and all the ten genotypes were used to construct the 1-genotype populations. For the 4-genotype and 8-genotype populations, each pot was grown with 16 ramets of four different genotypes and eight different genotypes, respectively. Each of these two diversity treatments was replicated five times, but the replication was at the diversity level rather than at the genotypic combination level (**Appendix Table S1**). This approach was commonly used when testing the effect of species or genotypic diversity (Wang et al., 2021; Begum et al., 2022; Huang et al., 2023). We also ensured that the frequency of occurrence of each genotype was the same.

For the 1-genotype treatment, populations of each of the ten genotypes were replicated nine times, resulting a total of 90 pots (populations). For the 4-genotype treatment, each of five 4-genotype populations was replicated three times, making 15 pots. Similarly, for the 8-genotype treatment, each of five 8-genotype populations was replicated three times, resulting in also 15 pots.

The experiment started on 30 August and ended on 30 October 2021, and was conducted in the greenhouse. During the experiment, the daily temperature in the greenhouse was controlled at 25.3°C. All pots were watered every two days, and were randomly repositioned three times during the experiment.

### 2.3 Measurements

We measured fluxes of CO_2_ and CH_4_ from each of the microcosms (the pots with the populations of *H. vulgaris* and the soil) between 09: 00 am and 11: 00 am every 10 days. For the 1-genotype treatment, we randomly selected three microcosms (from the nine microcosms) for each genotype to measure fluxes, and thus 30 microcosms in total. For the 4- and the 8-genoytype treatment, we randomly selected one microcosm (from the three microcosms) for each of the five genotype combinations of each treatment to measure fluxes, and thus ten microcosms in total.

To qualify CO_2_ and CH_4_ fluxes from the microcosms, gas concentration was determined with the Ultra-Portable Greenhouse Gas Analyzer (UGGA) (M-GGA-918, Los Gatos Research Corp. USA). During the measurement, we placed the transparent chamber (20 cm in inner diameter and 50 cm in inner height) in the microcosms and fitted it tightly to ensure that the chamber enclosed the plant and soils and sealed. Then we captured the greenhouse gas fluxes from the microcosms. Cumulative fluxes were calculated by multiplying the average of the fluxes of consecutive sampling days by the time interval between them. The positive value of fluxes means that emission is greater than absorption and is a carbon source; when flux is negative, it is a carbon sink.

The carbon dioxide equivalent (CO_2_-eq) is a standardized measurement unit that quantifies the effect of greenhouse gases, based on their reference to CO_2_ (Stocker, 2014; He et al., 2017). In our experiment, CO_2_-eq was defined as (He et al., 2017):


$$CO_{2}-eq=CO_{2}+(GWP_{M}\times M_{CH4})$$

where CO_2_ is the carbon dioxide flux (g CO_2_ m^-2^), M_CH4_ is the CH_4_ flux (g CH_4_ m^-2^) and GWP*_M_* is 25 (Stocker, 2014).

At the end of the experiment, we counted ramets of *H. vulgaris* and then harvested leaves, creeping stems, and root. We also measured leaf area (WinFOLIA Pro 2004a, Regent Instruments, Inc., QC, Canada) and total creeping stem length. All plant materials were sorted, oven-dried at 70°C for 72 h, and weighed.

### 2.4 Data analysis

We used the additive partitioning method (Loreau and Hector, 2001) to calculate the biodiversity effect of aboveground biomass. The net diversity effect is defined as the difference between the observed yield of *H. vulgaris in* mixture populations and the expected yield (i.e., the product of the yield of genotypes and the proportion of planted in mixture). It can be partitioned into the selection and complementarity effects (Loreau and Hector, 2001):


Selection effect=N∗ cov(ΔRY,M)

Complementarity effect=N∗mean(ΔRY)∗mean(M)

where *N* is the number of genotypes in the mixture, Δ*RY* is the change in relative yield for genotypes in the mixture,*M* is the yield of genotypes in monoculture and cov(Δ*RY,M)*is covariance of Δ*RY* and *M*.

Repeated-measures analysis of variance (ANOVA) was used to test for the effects on fluxes of CO_2_, CH_4_ and CO_2_-eq from the microcosms, with genotypes or genotypic diversity as a between-subject factor and the growth period as a within-subject factor. We used one-way ANOVA to examine the effect of genotype identity or genotypic diversity on plant growth (total biomass, aboveground biomass, belowground biomass, and leaf area) and cumulative fluxes of CO_2_, CH_4_ and CO_2_-eq from the microcosms and Duncan tests for multiple comparisons. Linear regression was used to examine the relationships of cumulative fluxes of CH_4_ and CO_2_ with total biomass, aboveground biomass, belowground biomass, and leaf area of *H. vulgaris*. Before analysis, all data were checked for normality and homogeneity of variance. Data of CH_4_ flux were logarithmically transformed to satisfy the assumptions of normality and homogeneity of variance. The analyses were conducted with SPSS 18.0 (SPSS Inc., Chicago, IL, U.S.A.). Effects were considered significant if *P<* 0.05. Partial Least Squares Path Modeling (PLSPM) was used to examine the direct and indirect effects of genotypic diversity of *H. vulgaris* on CO_2_ flux. PLSPM was conducted using the “plspm” R package (Luo et al., 2017).

## 3 Results

### 3.1 Genotypic differences in greenhouse gas fluxes

**Figure 1:**
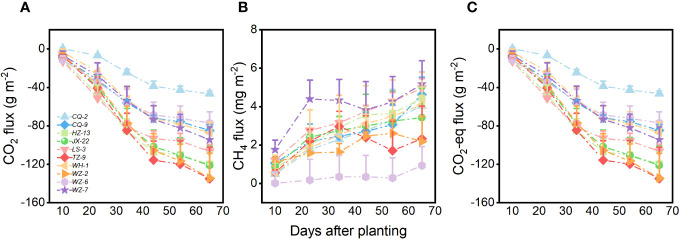
Fluxes of **(A)** CO_2_, **(B)** CH_4_ and **(C)** CO_2_ equivalent (CO_2_-eq) from the microcosms with populations consisting each of the ten genotypes of *H. vulgaris*. Vertical bars are standard errors.

Genotype of *H. vulgaris* had a significant effect on fluxes of CO_2_ and CO_2_-eq (**Table 1**). The values of the CO_2_ flux and CO_2_-eq flux from the microcosms were the smallest when they were planted with the genotype of *TZ-9* and the genotype of *WZ-2*, largest when they were planted with the genotype of *CQ-2*, and intermediate when they were planted with any other genotypes (**Figures 1A, C**). The growth of the *H. vulgaris* population was the largest when the population consisted of the genotype of *TZ-9* or the genotype of *LS-3*, smallest when it consisted of the genotype of *CQ-2*, and intermediate when it consisted of any of the other genotypes (**Appendix Figure S1**). Irrespective of the genotypes planted, the values of the CO_2_ flux and CO_2_-eq flux from the microcosms all decreased sharply during the experiment, but the differences between genotypes became larger with time (**Figures 1A, C**). However, genotype had no effect on the CH_4_ (**Table 1**; **Figure 1B**).

**Table 1:** ANOVAs of effects of genotype of *Hydrocotyle vulgaris*, growth period and their interaction on fluxes of CO_2_, CH_4_ and CO_2_ equivalent (CO_2_-eq) from the microcosms.

		Genotype (G)		Growth period (P)		G × P	
		*F*_9,20_	*P*	*F*_5,100_	*P*	*F*_45,100_	*P*
CO_2_ flux		2.51	**0.042**	405.80	**<0.001**	3.51	**0.002**
CH_4_ flux		1.11	0.401	27.69	**<0.001**	0.81	0.652
CO_2_-eq flux		2.51	**0.042**	405.22	**<0.001**	3.51	**0.002**

Values are in bold when *P*< 0.05.

**Figure 2:**
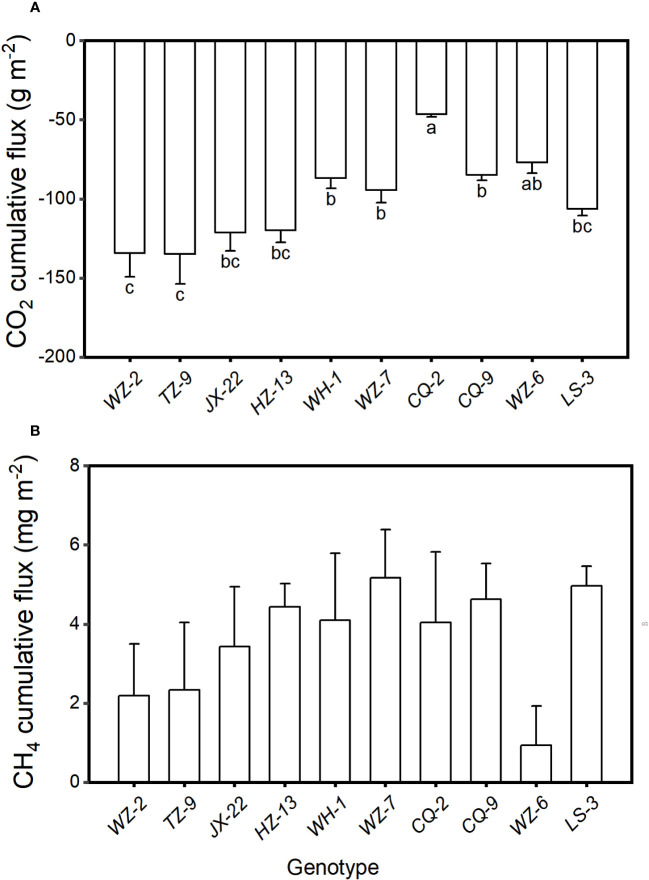
Cumulative fluxes of **(A)** CO_2_ and **(B)** CH_4_ from the microcosms with populations consisting each of the ten genotypes of *H. vulgaris*. Bars and vertical lines show means and SE. Bars sharing the same letter are statistically not different at *P* = 0.05. Different letters (a–c) at the end of bars indicate significant difference in each panel.

The cumulative CO_2_ flux varied significantly among the ten genotypes (*F*_9, 20 _= 2.77, *P* = 0.028). The cumulative flux of CO_2_ from the microcosms were the lowest when they were planted with the genotype of *TZ-9* (-134.87 ± 18.83 g m^-2^, mean ± SE) and the genotype of *WZ-2* (-134.41 ± 14.98 g m^-2^), the highest when they were planted with the genotype *CQ-2* (-46.51 ± 1.62 g m^-2^), and intermediated when they were planted with other genotypes (**Figure 2A**). However, the cumulative flux of CH_4_ did not differ significantly among different genotypes (**Figure 2B**, *F*_9, 20 _= 1.14, *P* = 0.383).

### 3.2 Effects of genotypic diversity on greenhouse gas fluxes

**Figure 3:**
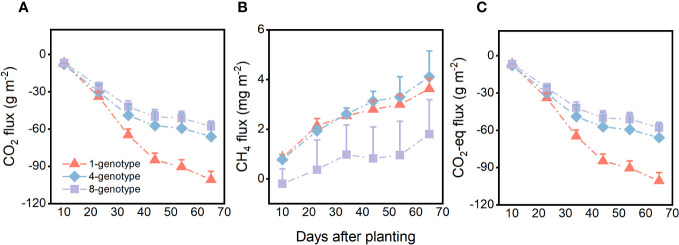
Effects of genotypic diversity of *H. vulgaris* and growth period on fluxes of **(A)** CO_2_, **(B)** CH_4_ and **(C)** CO_2_ equivalent (CO_2_-eq) from the microcosms. Vertical bars are standard errors.

**Figure 4:**
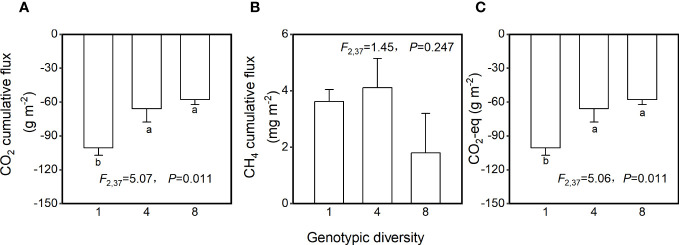
Effects of genotypic diversity of *H. vulgaris* on cumulative fluxes of **(A)** CO_2_, **(B)** CH_4_ and **(C)** CO_2_-eq from the microcosms. Bars and vertical lines show means and SE. F, *P* and degrees of freedom of one-way ANOVAs are given. Different letters (a–b) at the end of bars indicate significant difference in each panel.

Genotypic diversity of *H. vulgaris* had a significant effect on fluxes of CO_2_ and CO_2_-eq (**Table 2**). The CO_2_ flux and CO_2_-eq from the microcosms with the 1-genotype populations of *H. vulgaris* were significantly higher (i.e., values were more negative) than those with the 4- and 8-genotype populations, and such an effect increased significantly with increasing the growth period (significant interaction effect in **Table 2**; **Figures 3A, C**). Consequently, cumulative fluxes of CO_2_ (*F*_2, 37 _= 5.07, *P* = 0.011; **Figure 4A**) and CO_2_-eq (*F*_2, 37 _= 5.07, *P* = 0.011; **Figure 4C**) from the microcosms with the 1-genotype populations were significantly higher than those from the microcosms with the 4-and 8-genotype populations. While the CH_4_ flux increased significantly with increasing the growth period, it was not significantly influenced by genotypic diversity of *H. vulgaris* (**Table 2**, **Figure 3B**). Consequently, genotypic diversity did not significantly affect the cumulative flux of CH_4_ (*F*_2, 37 _ =1.45, *P* = 0.247; **Figure 4B**).

**Table 2:** ANOVAs of effects of genotypic diversity of *Hydrocotyle vulgaris*, growth period and their interaction on fluxes of CO_2_, CH_4_ and CO_2_ equivalent (CO_2_-eq) from the microcosms.

		Genotypic diversity (G)		Growth period (P)		G × P	
		*F*_2,37_	*P*	*F*_5,185_	*P*	*F*_10,185_	*P*
CO_2_ flux		**3.66**	**0.035**	93.93	**<0.001**	6.53	**0.001**
CH_4_ flux		2.34	0.111	19.18	**<0.001**	0.52	0.689
CO_2_-eq flux		**3.65**	**0.036**	93.75	**<0.001**	6.53	**0.001**

Values are in bold when *P*< 0.05.

### 3.3 Relationships between gas fluxes and plant growth

**Figure 5:**
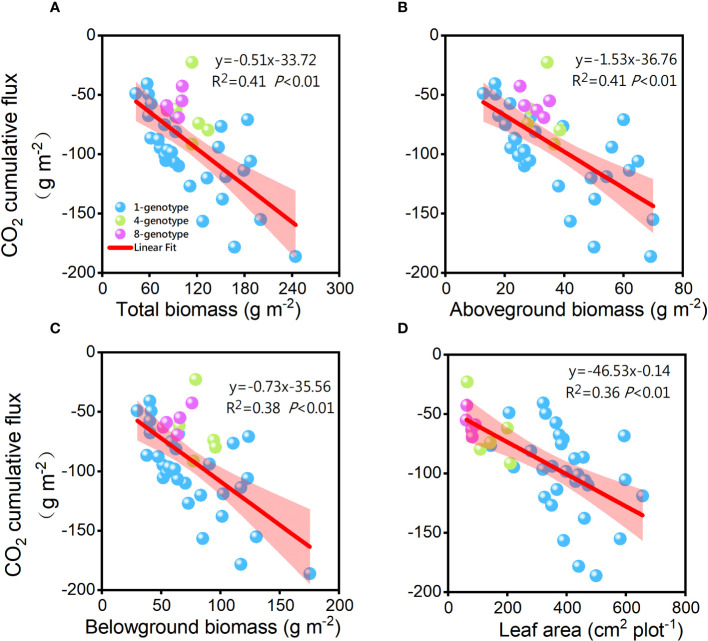
Relationships of the cumulative CO_2_ flux with **(A)** total biomass, **(B)** abovegroud biomass, **(C)** belowground biomass and **(D)** leaf area of the *H. vulgaris* populations. F-, R2-, and p-values obtained from linear regressions are also presented.

The cumulative CO_2_ flux was significantly negatively related to total biomass (R^2 ^= 0.41, *P<* 0.01), aboveground biomass (R^2 ^= 0.41, *P<* 0.01), belowground biomass (R^2 ^= 0.38, *P<* 0.01), and total leaf area (R^2 ^= 0.34, *P<* 0.01) of the *H. vulgaris* populations (**Figure 5**). However, the cumulative CH_4_ flux was not significantly related to total biomass (R^2^< 0.01, *P* = 0.980), aboveground biomass (R^2^< 0.01, *P* = 0.695), belowground biomass (R^2^< 0.01, *P* = 0.878) or leaf area (R^2 ^= 0.01, *P* = 0.520) of the *H. vulgaris* populations. The cumulative CO_2_ flux was not significantly related to any of the soil properties (**Appendix Figure S2**).

## 4 Discussion

We found that both genotypic identity and genotypic diversity of *H. vulgaris* influenced CO_2_ flux and CO_2_-eq from the plant-soil systems, suggesting that intraspecific variations of the same species can influence greenhouse gas fluxes. However, values of CO_2_ flux and CO_2_-eq from the microcosms were all negative, regardless of genotypic identity and diversity of *H. vulgaris*. This result suggests that photosynthesis was greater than respiration and CH_4_ emission (Fabre et al., 2020; Augusto and Boča, 2022).

CO_2_ flux varies among plant species (Priault et al., 2009). Our study shows that CO_2_ flux differs among genotypes, although the variation is less pronounced compared with plant species (Hughes et al., 2008; Crawford and Rudgers, 2012). Previous research has demonstrated the different traits of genotypic identity including growth rate (Poorter et al., 2005), respiration (Bulut et al., 2023), photosynthesis (Blackman et al., 2016) and related leaf traits (leaf nitrogen, leaf life, specific leaf area, etc.) (Grady et al., 2013). Our study similarly demonstrated significant differences of multiple traits among different genotypes, such as aboveground and belowground biomass, total biomass, and leaf area (**Appendix Figure S1**). The genotypes (*TZ-9* from Taizhou and *WZ-2* from Wenzhou) with significant carbon sequestration capacity had better population performance (**Appendix Figure S1**), indicating that these variations might account for the observed differences in CO_2_ flux. Moreover, previous studies have shown that genotypic identity may result in changes of root exudates production (Mönchgesang et al., 2016; Semchenko et al., 2018; Sun et al., 2021), and the microbial communities (Eck et al., 2019; Fernández et al., 2020; Begum et al., 2022). These alterations not only affect soil organic matter input, but also influence soil microbial activity (Raaijmakers et al., 2009). However, we did not find the effect of soil properties on CO_2_ flux, which may be due to the fact that we measured only a limited number of soil properties (i.e., soil moistures, soil total nitrogen, soil ammonium and nitrate nitrogen) and also due to the short experimental period (60 days), so that the effect of the plant on the soil was not enough to affect the CO_2_ flux (**Appendix Figure S2**). As a result, genotypic identity may impact plant photosynthesis and respiration rates, ultimately resulting in differences in CO_2_ flux (Mo et al., 2015; Gao et al., 2018; de Vries et al., 2019).

Surprisingly, the microcosms with the 1-genotype populations of *H. vulgaris*, had stronger carbon absorption capacity than those with the 4- and 8-genotype populations, suggesting that genotypic diversity of the same species can influence greenhouse gas flux. This might be because genotypic diversity has a negative effect on primary productivity and morphological traits of our plant communities (Adler et al., 2011). Previous studies have shown that genotypic diversity can increase population biomass (Hughes and Stachowicz, 2009), or has no effect or a negative effect on primary productivity (Fridley and Grime, 2010; Crawford and Rudgers, 2012). Such an inconstancy might be due to differences in environments, genotypes competition and resource availability (Fridley, 2002; Wang et al., 2019). In this experiment, the stolon of *H. vulgaris*, as stoloniferous plant, was restricted by the space of the experimental container during clonal reproduction, and the competition among genotypes increased. On the other hand, the more similar the phylogeny and the more overlapping the functional traits, the more inclined to increase competition among species and genotypes through selection effects (Venail and Vives, 2013; Huang et al., 2020). We hypothesized that although there were differences among genotypes, with the increase of genotypes, the probability of niche overlap increased, and the limited complementarity was more due to competition among *H. vulgaris* (**Appendix Figure S3**). Intense competition and resource constraints resulted in less biomass and leaf area, which was related to CO_2_ flux. We did not list all possible genotypic combinations, but there may be differences between genotypic combinations, and further studies can be designed to explore the underlying mechanisms.

Our findings revealed a negative correlation between CO_2_ flux and both biomass and leaf area of *H. vulgaris*, which is consistent with previous studies (Moore et al., 2002; Larmola et al., 2003). This relationship can be largely explained by the fact that CO_2_ assimilation is highly dependent on a plant’s photosynthetic potential and aboveground biomass (Wohlfahrt et al., 2008; Han et al., 2013), while leaf area is indicative of its photosynthetic capacity (Kaitaniemi, 2007; Lusk et al., 2019). Although studies have shown that biomass is also a predictor of CO_2_ flux (Emery and Fulweiler, 2014), in our experiment, we observed a higher rate of photosynthesis than respiration. As aboveground biomass and leaf area increased, net photosynthesis strengthened, leading to a decline in CO_2_ flux that ultimately became increasingly negative.

We found no significant relationship between CH_4_ flux and biomass, which is consistent with previous studies (Koelbener et al., 2010; Mo et al., 2015; Gao et al., 2018). As CH_4_ is produced by methanogens and then emitted through plants or soil (Zhang Y. et al., 2022), it is likely that biomass is not the sole predictor of CH_4_ production (Gong et al., 2021). Furthermore, we observed no significant differences in CH_4_ flux across genotypes in *H. vulgaris*. One possible explanation is that variation in genotypic identity of *H. vulgaris* may not strongly influence methanogen abundance or activity similarly to how specific identities do (Berg and Smalla, 2009; Edwards et al., 2015; Liechty et al., 2020). Additionally, CH_4_ flux may be mediated by processes related to transport from the soil to atmosphere (Ding et al., 2005; Bhullar et al., 2013; Andresen et al., 2017). However, the development of aerenchyma tissues and lacunae proportions in shoots and roots may not differ significantly among different genotypes of *H. vulgaris* (Ding et al., 2005). Consequently, limited trait variability across genotypes may also contribute to the lack of difference in CH_4_ flux of genotypic diversity observed in our experiment (Sutton Grier and Megonigal, 2011).

In conclusion, our results provide evidence that genotypic identity and diversity of the same species can influence greenhouse gas fluxes from plant-soil systems. These findings highlight the importance of intraspecific variation in mediating greenhouse gas fluxes and suggest that intraspecific variation should be considered when modelling regional and global patterns of greenhouse gas fluxes. One caveat is that this study was conducted in microcosms so that its reality is limited. Future studies could be designed to test how genotypic diversity can mediate greenhouse gas in field conditions.

## Data availability statement

The raw data supporting the conclusions of this article will be made available by the authors, without undue reservation.

## Author contributions

J-TZ: Writing – original draft. WX: Investigation, Writing – review & editing. J-QG: Writing – review & editing, Conceptualization. Q-WL: Investigation, Writing – review & editing. W-HY: Investigation, Writing – review & editing. F-HY: Conceptualization, Writing – review & editing.

## Supplementary material

Supplementary Figure 1 The total biomass, aboveground biomass, belowground biomass and leaf area of the ten genotypes of *H. vulgaris*. Bars and vertical lines show means and SE. Bars sharing the same letter are statistically not different at *P* = 0.05.

Supplementary Figure 2 Structure equation model showing the direct and indirect effects of genotypic diversity of *H. vulgaris* on CO_2_ flux. Red and blue arrows reflect positive and negative path ways respectively. Numbers along the arrows, as well as the width of the arrows, indicate standardized path coefficients. Significance levels of each predictor: **P*< 0.05, ***P*< 0.01, ****P*< 0.01.

Supplementary Figure 3 Net diversity effect, complementarity effect, selection effect on the aboveground biomass for 4- and 8-genotype populations of *H. vulgaris*. Bars and vertical lines show means and SE. Difference of values between 4- and 8-genotype populations were tested by independent samples t-test. * indicates the significance at *P*< 0.05.

## References

[B1] Abbott J. M. Stachowicz J. J. (2016). The relative importance of trait vs. genetic differentiation for the outcome of interactions among plant genotypes. Ecology 97, 84–94. doi: 10.1890/15-0148.1 27008778

[B2] Adler P. B. Seabloom E. W. Borer E. T. Hillebrand H. Hautier Y. Hector A.. (2011). Productivity is a poor predictor of plant species richness. Science 333, 1750–1753. doi: 10.1126/science.1204498 21940895

[B3] Adomako M. O. Ning L. Tang M. Du D. L. van Kleunen M. Yu F. H. (2019). Diversity- and density-mediated allelopathic effects of resident plant communities on invasion by an exotic plant. Plant Soil 440, 581–592. doi: 10.1007/s11104-019-04123-9

[B4] Andresen C. G. Lara M. J. Tweedie C. E. Lougheed V. L. (2017). Rising plant-mediated methane emissions from arctic wetlands. Glob. Change Biol. 23, 1128–1139. doi: 10.1111/gcb.13469 27541438

[B5] Augusto L. Boča A. (2022). Tree functional traits, forest biomass, and tree species diversity interact with site properties to drive forest soil carbon. Nat. Commun. 13, 1097. doi: 10.1038/s41467-022-28748-0 35233020 PMC8888738

[B6] Begum G. Gao J. Q. Song M. H. Xue W. Yu F. H. (2022). Direct and legacy effects of genotypic diversity on population performance of *Hydrocotyle vulgaris* . Ecol. Indic. 144, 109570. doi: 10.1016/j.ecolind.2022.109570

[B7] Begum G. Gao J. Q. Xue W. Yu F. H. (2023). Plant-soil feedbacks in *Hydrocotyle vulgaris*: Genotypic differences and relations to functional traits. Ecol. Indic. 146, 109766. doi: 10.1016/j.ecolind.2022.109766

[B8] Berg G. Smalla K. (2009). Plant species and soil type cooperatively shape the structure and function of microbial communities in the rhizosphere. FEMS Microbiol. Ecol. 68, 1–13. doi: 10.1111/j.1574-6941.2009.00654.x 19243436

[B9] Bhullar G. S. Edwards P. J. Olde Venterink H. (2013). Variation in the plant-mediated methane transport and its importance for methane emission from intact wetland peat mesocosms. J. Plant Ecol. 6, 298–304. doi: 10.1093/jpe/rts045

[B10] Blackman C. J. Aspinwall M. J. Resco de Dios V. Smith R. A. Tissue D. T. (2016). Leaf photosynthetic, economics and hydraulic traits are decoupled among genotypes of a widespread species of eucalypt grown under ambient and elevated CO_2_ . Funct. Ecol. 30, 1491–1500. doi: 10.1111/1365-2435.12661

[B11] Bulut M. Alseekh S. Fernie A. R. (2023). Natural variation of respiration-related traits in plants. Plant Physiol. 191, 2120–2132. doi: 10.1093/plphys/kiac593 36546766 PMC10069898

[B12] Burrill H. M. Wang G. Bever J. D. (2023). Rapid differentiation of soil and root microbiomes in response to plant composition and biodiversity in the field. ISME Commun. 3, 31. doi: 10.1038/s43705-023-00237-5 37076650 PMC10115818

[B13] Cardinale B. J. Duffy J. E. Gonzalez A. Hooper D. U. Perrings C. Venail P.. (2012). Biodiversity loss and its impact on humanity. Nature 486, 59–67. doi: 10.1038/nature11148 22678280

[B14] Cook-Patton S. C. McArt S. H. Parachnowitsch A. L. Thaler J. S. Agrawal A. A. (2011). A direct comparison of the consequences of plant genotypic and species diversity on communities and ecosystem function. Ecology 92, 915–923. doi: 10.1890/10-0999.1 21661554

[B15] Crawford K. M. Rudgers J. A. (2012). Plant species diversity and genetic diversity within a dominant species interactively affect plant community biomass. J. Ecol. 100, 1512–1521. doi: 10.1111/j.1365-2745.2012.02016.x

[B16] de Vries F. T. Williams A. Stringer F. Willcocks R. McEwing R. Langridge H.. (2019). Changes in root-exudate-induced respiration reveal a novel mechanism through which drought affects ecosystem carbon cycling. New Phytol. 224, 132–145. doi: 10.1111/nph.16001 31218693 PMC6771481

[B17] Ding W. Cai Z. Tsuruta H. (2005). Plant species effects on methane emissions from freshwater marshes. Atmospheric Environ. 39, 3199–3207. doi: 10.1016/j.atmosenv.2005.02.022

[B18] Dong B. C. Wang J. Z. Liu R. H. Zhang M. X. Luo F. L. Yu F. H. (2015). Soil heterogeneity affects ramet placement of *Hydrocotyle vulgaris* . J. Plant Ecol. 8, 91–100. doi: 10.1093/jpe/rtu003

[B19] Eck J. L. Stump S. M. Delavaux C. S. Mangan S. A. Comita L. S. (2019). Evidence of within-species specialization by soil microbes and the implications for plant community diversity. Proc. Natl. Acad. Sci. U.S.A. 116, 7371–7376. doi: 10.1073/pnas.1810767116 30842279 PMC6462086

[B20] Edwards J. Johnson C. Santos-Medellín C. Lurie E. Podishetty N. K. Bhatnagar S.. (2015). Structure, variation, and assembly of the root-associated microbiomes of rice. Proc. Natl. Acad. Sci. U.S.A. 112, E911–E920. doi: 10.1073/pnas.1414592112 25605935 PMC4345613

[B21] Emery H. E. Fulweiler R. W. (2014). *Spartina alterniflora* and invasive *Phragmites australis* stands have similar greenhouse gas emissions in a New England marsh. Aquat. Bot. 116, 83–92. doi: 10.1016/j.aquabot.2014.01.010

[B22] Fabre D. Dingkuhn M. Yin X. Clément Vidal A. Roques S. Soutiras A.. (2020). Genotypic variation in source and sink traits affects the response of photosynthesis and growth to elevated atmospheric CO_2_ . Plant Cell Environ. 43, 579–593. doi: 10.1111/pce.13693 31961455

[B23] Fan X. Du Y. Luo B. Han W. Niu S. Gu W.. (2021). Increasing plant diversity to mitigate net greenhouse effect of wastewater treatment in floating constructed wetlands. J. Clean. Prod. 314, 127955. doi: 10.1016/j.jclepro.2021.127955

[B24] Fang J. (2010). Soils emitting more carbon dioxide. Nature. doi: 10.1038/news.2010.147

[B25] Fernández N. V. Marchelli P. Tenreiro R. Chaves S. Fontenla S. B. (2020). Are the rhizosphere fungal communities of *Nothofagus alpina* established in two different environments influenced by plant genetic diversity? For. Ecol. Manage. 473, 118269. doi: 10.1016/j.foreco.2020.118269

[B26] Fridley J. D. (2002). Resource availability dominates and alters the relationship between species diversity and ecosystem productivity in experimental plant communities. Oecologia 132, 271–277. doi: 10.1007/s00442-002-0965-x 28547362

[B27] Fridley J. D. Grime J. P. (2010). Community and ecosystem effects of intraspecific genetic diversity in grassland microcosms of varying species diversity. Ecology 91, 2272–2283. doi: 10.1890/09-1240.1 20836449

[B28] Gao J. Q. Duan M. Y. Zhang X. Y. Li Q. W. Yu F. H. (2018). Effects of frequency and intensity of drying-rewetting cycles on *Hydrocotyle vulgaris* growth and greenhouse gas emissions from wetland microcosms. Catena 164, 44–49. doi: 10.1016/j.catena.2018.01.006

[B29] Gong Y. Wu J. Le T. B. (2021). Counteractions between biotic and abiotic factors on methane dynamics in a boreal peatland: Vegetation composition change vs warming and nitrogen deposition. Geoderma 395, 115074. doi: 10.1016/j.geoderma.2021.115074

[B30] Grady K. C. Laughlin D. C. Ferrier S. M. Kolb T. E. Hart S. C. Allan G. J.. (2013). Conservative leaf economic traits correlate with fast growth of genotypes of a foundation riparian species near the thermal maximum extent of its geographic range. Funct. Ecol. 27, 428–438. doi: 10.1111/1365-2435.12060

[B31] Han W. Luo G. Luo B. Yu C. Wang H. Chang J.. (2019). Effects of plant diversity on greenhouse gas emissions in microcosms simulating vertical constructed wetlands with high ammonium loading. J. Environ. Sci. 77, 229–237. doi: 10.1016/j.jes.2018.08.001 30573087

[B32] Han G. X. Yang L. Yu J. Wang G. Mao P. Gao Y. (2013). Environmental controls on net ecosystem CO_2_ exchange over a reed (*Phragmites australis*) wetland in the Yellow River Delta, China. Estuaries Coast. 36, 401–413. doi: 10.1007/s12237-012-9572-1

[B33] He Y. Zhou X. Jiang L. Li M. Du Z. Zhou G.. (2017). Effects of biochar application on soil greenhouse gas fluxes: a meta-analysis. Glob. Change Biol. Bioenergy 9, 743–755. doi: 10.1111/gcbb.12376

[B34] Huang M. Liu X. Cadotte M. W. Zhou S. (2020). Functional and phylogenetic diversity explain different components of diversity effects on biomass production. Oikos 129, 1185–1195. doi: 10.1111/oik.07032

[B35] Huang L. Yao S. M. Jin Y. Xue W. Yu F. H. (2023). Co-contamination by heavy metal and organic pollutant alters impacts of genotypic richness on soil nutrients. Front. Plant Sci. 14. doi: 10.3389/fpls.2023.1124585 PMC9909551 36778695

[B36] Huang L. Yu M. F. Hu J. N. Sheng W. J. Xue W. Yu F. H. (2022). Density alters impacts of genotypic evenness on productivity in an experimental plant population. Front. Plant Sci. 13. doi: 10.3389/fpls.2022.915812 PMC9197231 35712564

[B37] Hughes A. R. Inouye B. D. Johnson M. T. Underwood N. Vellend M. (2008). Ecological consequences of genetic diversity. Ecol. Lett. 11, 609–623. doi: 10.1111/j.1461-0248.2008.01179.x 18400018

[B38] Hughes A. R. Stachowicz J. J. (2009). Ecological impacts of genotypic diversity in the clonal seagrass *Zostera marina* . Ecology 90, 1412–1419. doi: 10.1890/07-2030.1 19537560

[B39] Jiang M. Yang X. Wang T. Xu Y. Dong K. He L.. (2021). A direct comparison of the effects and mechanisms between species richness and genotype richness in a dominant species on multiple ecosystem functions. Ecol. Evol. 11, 14125–14134. doi: 10.1002/ece3.8125 34707845 PMC8525171

[B40] Johnson D. Anderson I. C. Williams A. Whitlock R. Grime J. P. (2010). Plant genotypic diversity does not beget root-fungal species diversity. Plant Soil 336, 107–111. doi: 10.1007/s11104-010-0452-9

[B41] Kaitaniemi P. (2007). Consequences of variation in tree architecture and leaf traits on light capture and photosynthetic nitrogen use efficiency in mountain birch. Arct. Antarct. Alp. Res. 39, 258–267. doi: 10.1657/1523-0430(2007)39[258:COVITA]2.0.CO;2

[B42] Koelbener A. Ström L. Edwards P. J. Olde Venterink H. (2010). Plant species from mesotrophic wetlands cause relatively high methane emissions from peat soil. Plant Soil 326, 147–158. doi: 10.1007/s11104-009-9989-x

[B43] Larmola T. Alm J. Juutinen S. Martikainen P. J. Silvola J. (2003). Ecosystem CO_2_ exchange and plant biomass in the littoral zone of a boreal eutrophic lake. Freshw. Biol. 48, 1295–1310. doi: 10.1046/j.1365-2427.2003.01079.x

[B44] Latta L. C. Baker M. Crowl T. Jacob Parnell J. Weimer B. DeWald D. B.. (2011). Species and genotype diversity drive community and ecosystem properties in experimental microcosms. Ecol. Evol. 25, 1107–1125. doi: 10.1007/s10682-010-9457-3

[B45] Liechty Z. Santos Medellín C. Edwards J. Nguyen B. Mikhail D. Eason S.. (2020). Comparative analysis of root microbiomes of rice cultivars with high and low methane emissions reveals differences in abundance of methanogenic archaea and putative upstream fermenters. mSystems 5, e00897–e00819. doi: 10.1128/mSystems.00897-19 PMC7029222 32071162

[B46] Loreau M. Hector A. J. N. (2001). Partitioning selection and complementarity in biodiversity experiments. Nature 412, 72–76. doi: 10.1038/35083573 11452308

[B47] Luo B. Du Y. Han W. Geng Y. Wang Q. Duan Y.. (2020). Reduce health damage cost of greenhouse gas and ammonia emissions by assembling plant diversity in floating constructed wetlands treating wastewater. J. Clean. Prod. 244, 118927. doi: 10.1016/j.jclepro.2019.118927

[B48] Luo Z. Feng W. Luo Y. Baldock J. Wang E. (2017). Soil organic carbon dynamics jointly controlled by climate, carbon inputs, soil properties and soil carbon fractions. Glob Change Biol. 23, 4430–4439. doi: 10.1111/gcb.13767 28544252

[B49] Lusk C. H. Grierson E. R. P. Laughlin D. C. (2019). Large leaves in warm, moist environments confer an advantage in seedling light interception efficiency. New Phytol. 223, 1319–1327. doi: 10.1111/nph.15849 30985943

[B50] Mo Y. Deng Z. H. Gao J. Q. Guo Y. X. Yu F. H. (2015). Does richness of emergent plants affect CO_2_ and CH_4_ emissions in experimental wetlands? Freshw. Biol. 60, 1537–1544. doi: 10.1111/fwb.12586

[B51] Mönchgesang S. Strehmel N. Schmidt S. Westphal L. Taruttis F. Müller E.. (2016). Natural variation of root exudates in *Arabidopsis* thaliana-linking metabolomic and genomic data. Sci. Rep. 6, 29033. doi: 10.1038/srep29033 27363486 PMC4929559

[B52] Moore T. R. Bubier J. L. Frolking S. E. Lafleur P. M. Roulet N. T. (2002). Plant biomass and production and CO_2_ exchange in an ombrotrophic bog. J. Ecol. 90, 25–36. doi: 10.1046/j.0022-0477.2001.00633.x

[B53] Poorter H. van Rijn C. P. E. Vanhala T. K. Verhoeven K. J. F. de Jong Y. E. M. Stam P.. (2005). A genetic analysis of relative growth rate and underlying components in *Hordeum spontaneum* . Oecologia 142, 360–377. doi: 10.1007/s00442-004-1705-1 15655691

[B54] Priault P. Wegener F. Werner C. (2009). Pronounced differences in diurnal variation of carbon isotope composition of leaf respired CO_2_ among functional groups. New Phytol. 181, 400–412. doi: 10.1111/j.1469-8137.2008.02665.x 19121035

[B55] Raaijmakers J. M. Paulitz T. C. Steinberg C. Alabouvette C. Moënne Loccoz Y. (2009). The rhizosphere: a playground and battlefield for soilborne pathogens and beneficial microorganisms. Plant Soil 321, 341–361. doi: 10.1007/s11104-008-9568-6

[B56] Ritchie H. Roser M. Rosado P. (2020). CO_2_ and Greenhouse Gas Emissions (OurWorldInData.org). Available at: https://ourworldindata.org/CO2-and-greenhouse-gas-emissions.

[B57] Semchenko M. Lepik A. Abakumova M. Zobel K. (2018). Different sets of belowground traits predict the ability of plant species to suppress and tolerate their competitors. Plant Soil 424, 157–169. doi: 10.1007/s11104-017-3282-1

[B58] Semchenko M. Xue P. Leigh T. (2021). Functional diversity and identity of plant genotypes regulate rhizodeposition and soil microbial activity. New Phytol. 232, 776–787. doi: 10.1111/nph.17604 34235741

[B59] Si C. Alpert P. Zhang J. F. Lin J. Wang Y. Y. Hong M. M.. (2020). Capacity for clonal integration in introduced versus native clones of the invasive plant *Hydrocotyle vulgaris* . Sci. Total Environ. 745, 141056. doi: 10.1016/j.scitotenv.2020.141056 32717606

[B60] Stocker T. (2014). Climate change 2013: the physical science basis: Working group I contribution to the fifth assessment report of the Intergovernmental panel on climate change (Cambridge: Cambridge University Press). doi: 10.1017/CBO9781107415324

[B61] Sun L. Ataka M. Han M. Han Y. Gan D. Xu T.. (2021). Root exudation as a major competitive fine-root functional trait of 18 coexisting species in a subtropical forest. New Phytol. 229, 259–271. doi: 10.1111/nph.16865 32772392

[B62] Sutton Grier A. E. Megonigal J. P. (2011). Plant species traits regulate methane production in freshwater wetland soils. Soil Biol. Biochem. 43, 413–420. doi: 10.1016/j.soilbio.2010.11.009

[B63] van der Plas F. (2019). Biodiversity and ecosystem functioning in naturally assembled communities. Biol. Rev. 94, 1220–1245. doi: 10.1111/brv.12499 30724447

[B64] Venail P. A. Vives M. J. (2013). Phylogenetic distance and species richness interactively affect the productivity of bacterial communities. Ecology 94, 2529–2536. doi: 10.1890/12-2002.1 24400504

[B65] Wang Y. J. Chen D. Yan R. Yu F. H. van Kleunen M. (2019). Invasive alien clonal plants are competitively superior over co-occurring native clonal plants. Perspect. Plant Ecol. Evol. Syst. 40, 125484. doi: 10.1016/j.ppees.2019.125484

[B66] Wang X. Y. Ge Y. Gao S. Chen T. Wang J. Yu F. H. (2021). Evenness alters the positive effect of species richness on community drought resistance *via* changing complementarity. Ecol. Indic. 133, 108464. doi: 10.1016/j.ecolind.2021.108464

[B67] Wang M. Z. Li H. L. Li J. M. Yu F. H. (2020). Correlations between genetic, epigenetic and phenotypic variation of an introduced clonal herb. Heredity 124, 146–155. doi: 10.1038/s41437-019-0261-8 31431739 PMC6906319

[B68] Wang M. Z. Li H. L. Tang M. Yu F. H. (2022). DNA methylation correlates with eesponses of experimental *Hydrocotyle vulgaris* populations to different flood regimes. Front. Plant Sci. 13. doi: 10.3389/fpls.2022.831175 PMC8940293 35330870

[B69] Wohlfahrt G. Anderson Dunn M. Bahn M. Balzarolo M. Berninger F. Campbell C.. (2008). Biotic, abiotic, and management controls on the net ecosystem CO_2_ exchange of european mountain grassland ecosystems. Ecosystems 11, 1338–1351. doi: 10.1007/s10021-008-9196-2

[B70] Xue W. Huang L. Sheng W. J. Zhu J. T. Li S. Q. Yu F. H. (2022). Contrasting effects of plant-soil feedbacks on growth and morphology of physically-connected daughter and mother ramets in two clonal plants. Plant Soil 472, 479–489. doi: 10.1007/s11104-021-05266-4

[B71] Xue W. Yao S. M. Huang L. Roiloa S. R. Ji B. M. Yu F. H. (2021). Current plant diversity but not its soil legacy influences exotic plant invasion. J. Plant Ecol. 15, 639–649. doi: 10.1093/jpe/rtab065

[B72] Yuan J. Ding W. Liu D. Kang H. Freeman C. Xiang J.. (2015). Exotic *Spartina alterniflora* invasion alters ecosystem–atmosphere exchange of CH_4_ and N_2_O and carbon sequestration in a coastal salt marsh in China. Glob. Chang Biol. 21, 1567–1580. doi: 10.1111/gcb.12797 25367159

[B73] Zhang L. Alpert P. Yu F. H. (2022). Nutrient foraging ability promotes intraspecific competitiveness in the clonal plant *Hydrocotyle vulgaris* . Ecol. Indic. 138, 108862. doi: 10.1016/j.ecolind.2022.108862

[B74] Zhang Y. Song C. Wang X. Chen N. Zhang H. Du Y.. (2022). Warming effects on the flux of CH_4_ from peatland mesocosms are regulated by plant species composition: Richness and functional types. Sci. Total Environ. 806, 150831. doi: 10.1016/j.scitotenv.2021.150831 34627884

