# Supplementary material for: Does genotypic diversity of Hydrocotyle vulgaris affect CO2 and CH4 fluxes?
Source: Front Plant Sci. 2023 Oct 9;14:1272313. doi: 10.3389/fpls.2023.1272313 (PMC10591177; doi:10.3389/fpls.2023.1272313)
Supplement: Supplementary file 1 [file Table_1.docx]

Table S1 Genotypic combination used for mixture populations. The black blocks represent the cases in which the genotype appears in the replicates.

|  | Mixture populations | | | | | | | | | |
| --- | --- | --- | --- | --- | --- | --- | --- | --- | --- | --- |
|  | 4-genotype richness | | | | | 8-genotype richness | | | | |
| *CQ-2* |  |  |  |  |  |  |  |  |  |  |
| *CQ-9* |  |  |  |  |  |  |  |  |  |  |
| *HZ-13* |  |  |  |  |  |  |  |  |  |  |
| *WZ-2* |  |  |  |  |  |  |  |  |  |  |
| *WZ-6* |  |  |  |  |  |  |  |  |  |  |
| *WZ-7* |  |  |  |  |  |  |  |  |  |  |
| *TZ-9* |  |  |  |  |  |  |  |  |  |  |
| *LS-3* |  |  |  |  |  |  |  |  |  |  |
| *JX-22* |  |  |  |  |  |  |  |  |  |  |
| *WH-1* |  |  |  |  |  |  |  |  |  |  |
