# Supplementary figures and images for: Does genotypic diversity of Hydrocotyle vulgaris affect CO2 and CH4 fluxes?
Source: Front Plant Sci. 2023 Oct 9;14:1272313. doi: 10.3389/fpls.2023.1272313 (PMC10591177; doi:10.3389/fpls.2023.1272313)

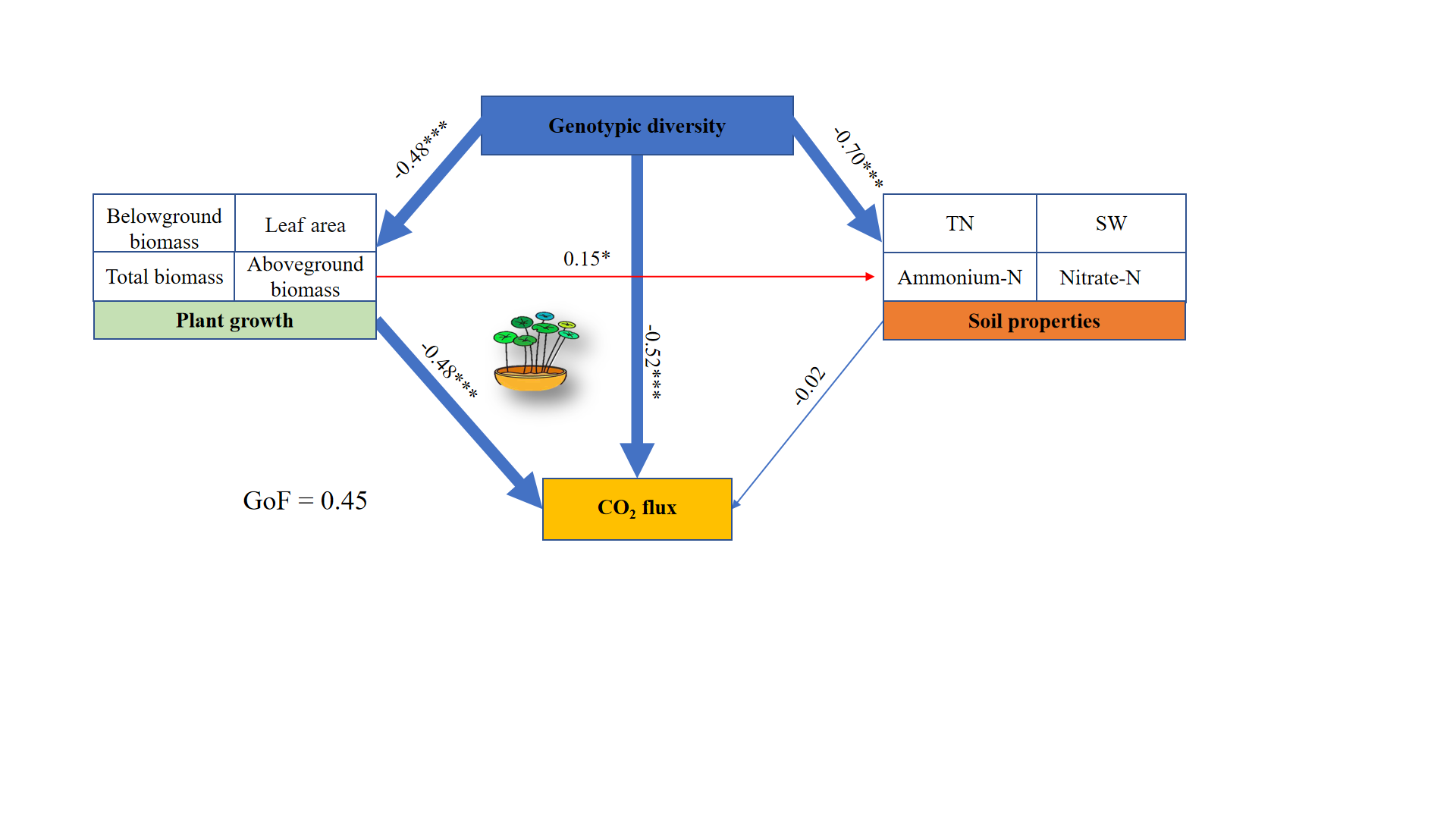

Supplement: Supplementary Figure 2 — Structure equation model showing the direct and indirect effects of genotypic diversity of H. vulgaris on CO2 flux. Red and blue arrows reflect positive and negative path ways respectively. Numbers along the arrows, as well as the width of the arrows, indicate standardized path coefficients. Significance levels of each predictor: *P< 0.05, **P< 0.01, ***P< 0.01. [file Image_2.tif]
